# A Novel* MLH1* Initiation Codon Mutation (c.3G>T) in a Large Chinese Lynch Syndrome Family with Different Onset Age and mRNA Expression Level

**DOI:** 10.1155/2018/1460835

**Published:** 2018-11-14

**Authors:** Yanni Zhang, Huishuang Chen, Zhiyu Peng, Santasree Banerjee, Wei Li, Zhaolong Zhao, Jianbin Sun, Jian Lv, Hui Huang, Ru Bai, Keke Lin, Zhongxin Li

**Affiliations:** ^1^Second Department of Surgery, Hebei Medical University Fourth Affiliated Hospital and Hebei Provincial Tumor Hospital, 12 Jiankang Road, Shijiazhuang, Hebei 050011, China; ^2^BGI Genomics, BGI-Shenzhen, Shenzhen 518083, China

## Abstract

Lynch syndrome is a genetically and clinically heterogeneous disorder; it is caused by a germline mutation in DNA mismatch repair (MMR) genes. Individuals with a heterozygous mutation in MLH1 have an increased risk for developing colorectal cancer. Here we described a 5-generation Chinese Lynch syndrome family with different severity and onset age. A novel heterozygous germline mutation (c.3G>T, p.Met1Ile) in* MLH1* gene was discovered by next generation sequencing. Our study also revealed by qPCR that the MLH1 mRNA expression in peripheral blood of patients in this family was remarkably lower than that of the unaffected carriers and non-carriers. The research results indicated that the mRNA expression level may provide predictive suggestions of treatment and management for carriers with the initiation codon mutation of* MLH1 *in this family. Further studies are undertaken in this family as well as other families with Lynch syndrome to interrogate the exact reasons affecting the MLH1 mRNA expression level and whether mRNA expression in peripheral blood could be a significant factor for early diagnosis and surveillance of Lynch syndrome.

## 1. Introduction

Lynch syndrome (LS), with an autosomal dominant mode of inheritance, is also known as hereditary nonpolyposis colorectal cancer (HNPCC) [OMIM#120435]. Some germline mutations in DNA mismatch repair (MMR) genes, such as* MLH1, MSH2*,*, MSH6*, and* PMS2*, are associated with Lynch syndrome. MMR genes play an important role in recognizing and repairing erroneous insertions, deletions, and other mutations that occur during DNA replication [1]. There are several other genes, such as EPCAM, MLH3, and PMS1, that may contribute to the mismatch repair process; these genes have been reported in some families with Lynch syndrome [2–4]. Lynch syndrome is a genetically and clinically heterogeneous disorder. Different types of pathogenic variants in different MMR genes may result in different cumulative risk, clinical phenotypes, and onset age at diagnosis. The same mutation in a family may have a wide variety of clinical phenotypes; this potentially complicates surveillance, counseling, diagnosis, and treatment strategies for mutation carriers.

Hereditary nonpolyposis colorectal cancer-2 (HNPCC2) is caused by the germline mutation of* MLH1* gene [5]. The cumulative risk of* MLH1* associated colorectal cancer for men and women is 75.4% and 76.9%, respectively [6]. The average age of diagnosis in HNPCC2 kindreds is 38.5 years, which is earlier than other types of HNPCC [7]. The cumulative 5-year survival following colorectal cancer in* MLH1* mutation carriers was 56.2% [8, 9]. A simple tool to predict the age of onset, severity, and survival rate of* MLH1* mutation carriers would be of prime clinical importance.

Previous studies of Lynch syndrome mainly focused on molecular genetic screening of the germline MMR genes, immunohistochemistry (IHC), and microsatellite instability (MSI) of the tumor tissue. However, germline mutation only provides a general lifetime risk for* MLH1* mutation carriers [6, 10]. The detection of tumor tissue can only be performed on cancer patients. There may be a distinct difference in severity and age of onset in this family which cannot be predicted by standard tests.

In this study, we investigate a Chinese family with Lynch syndrome for five generations. The patients of this family exhibited a significantly different onset age and severity. A novel nucleotide substitution (c.3G>T) in* MLH1* gene was detected from this family. We studied the* MLH1* mRNA level in the peripheral blood of the proband and his family members (including Lynch syndrome survivors) by real-time quantitative polymerase chain reaction (qPCR). Our goal was to search for a way to predict the manifestation and to provide individualized surveillance (such as routine colonoscopy and prophylactic removal) before the development of cancer for each* MLH1 *mutation carrier in this family.

## 2. Materials and Methods

### 2.1. Identification of the Novel* MLH1* Mutation

Because of different severity and onset age in this family, we performed target exome-based next generation sequencing on DNA samples of the proband and his father in order to exclude compound heterozygous mutations in the MMR genes [11].

### 2.2. Targeted Exome Based High Throughput Sequencing

In order to discover all the possible pathogenic mutations related to colorectal cancer in this family, 14 CRC related genes, including all the MMR genes, were selected from OMIM (http://www.ncbi.nlm.nih.gov/omim/). Roche NimbleGen's (Madison, USA) custom Sequence Capture Human Array covering the exonic sequences (except for the high repetitive regions of* STK11* exon1) and flanking 10bp was used. The library preparation was consistent with standard protocols. Enriched DNA samples were pooled and sequenced on Illumina HiSeq 2500 Analyzers (Illumina, San Diego, USA) to generate 90 bps of paired-end reads.

### 2.3. Variant Annotation and Interpretation

Illumina Pipeline software (version 1.3.4) was used to generate raw data. Clean reads were then generated from the raw reads following our filtering criteria. Sequence alignment of the clean data was performed using NextGENe software (SoftGenetics, State College, Pa). The reference was obtained from the NCBI, version GRCh37 (hg19). SNVs were called according to our previously reported method [12]. In order to detect large exonic deletions and duplications together, a coverage-based algorithm [13] was used in our interpretation.

### 2.4. Sanger Sequencing

Sanger sequencing was performed on the proband and his family members to verify the true positive of the identified mutation and to confirm the carriers. Primers designing, PCR amplification, and sequencing were carried out according to the standard protocols at www.impactjournals.com.

### 2.5. Real-Time Quantitative RT-PCR

We collected 5 mL peripheral blood samples from the proband, as well as 8 of his family members. These samples were stored in 10 mL vacuum tube containing 200 *μ*l of 0.5mol/L EDTA. Ficoll-Hypaque density gradient centrifugation was used to extract human peripheral blood mononuclear cells (PBMCs). TRIzol Reagent (Invitrogen) was used to extract total RNA from the PBMCs. The RNA concentration and purity were checked by OD A260/A280 (>1.8) and A260/A230 (>1.6). ABI High-Capacity cDNA reverse transcription kits were used to make reverse transcriptions of 2 *μ*g of total RNA. Synthesized cDNA was then stored at -20°C for later use. Using GAPDH as a housekeeping gene, the PCR was performed in triplicate using ABI vii7 real-time PCR detection system (Applied Biosystems) [14]. The nucleotide sequences of primers and probes were as follows: MLH1, forward primer is 5′-CCCAGGCCATTGTCACAGAG-3′, and reverse primer is 5′-TTTTTGGCAGCCACTTCAGC-3′; the forward primer of GAPDH is 5′-CTGCCAACGTGTCAGTGGTG-3′, and the reverse primer is 5′-TCAGTGTAGCCCAGGATGCC-3′. The relative MLH1 expression value was calculated via the 2−ΔΔCt method.

### 2.6. Mismatch Repair Protein Immunohistochemistry

Immunohistochemistry was performed on the proband's postoperative tissue in accordance with standard streptavidin–biotin–peroxidase procedures [15].

### 2.7. Statistics

All data were expressed as the* mean* ±* SEM*. Statistical comparisons were performed using Student's* t*-*test* (P values < 0.05 were considered to be statistically significant).

## 3. Results

### 3.1. The Family with Lynch Syndrome

**Figure 1:**
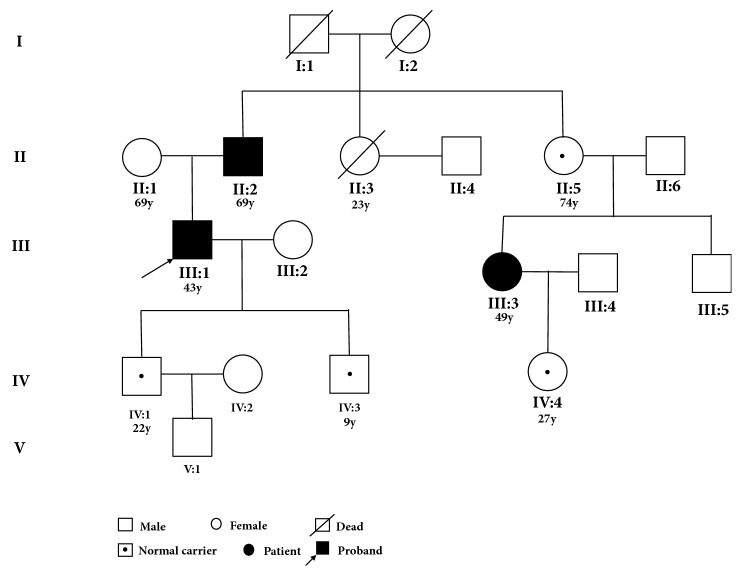
Pedigree of the Lynch family. The filled symbol indicates the affected individual, symbol with a dot belongs to carrier without having disease phenotype, square represents male and circle female, and symbol with a slash indicates deceased. Arrow indicates the proband. The age until this research was noted below the family members with detections.

Initially a 42-year-old male patient (III:1) was referred to the Second Department of Surgery, The Fourth Hospital of Hebei Medical University, and was clinically diagnosed with colorectal cancer. After a series of clinical detections of the proband and pedigree investigation of the 5-generation family (Figure 1), this family was diagnosed with Lynch syndrome according to Bethesda Guidelines which principally includes (1) colorectal cancer (CRC) diagnosed younger than 50 years, (2) CRC with the MSI-H histology, (3) one or more first-degree relatives diagnosed with a LS-related cancer before age of 50 years, and (4) at least one patient with CRC or other LS-related tumors diagnosed synchronously, or metachronously, regardless of age [16].

### 3.2. Family History and Clinical Description

In this 5-generation Chinese family, there are 3 family members clinically diagnosed with CRC (III:1, II:2, and III:4); 2 family members (I:2 and II:3) have died of intense abdominal pain at an early age without a timely diagnosis and treatment (Figure 1). They were supposed to die from colorectal cancer according to the descriptions of their family members. However, there is not definite diagnosis or autopsy attributed to the poor medical condition decades before in the rural areas. The other 12 family members in Figure 1 have received general physical examinations with no other types of cancer associated with Lynch syndrome found.

### 3.3. II-2: The Proband's Father

At the time of study, the proband's father was 62 years old. He developed 2 colorectal carcinomas metachronously: one in his transverse colon at the age of 27 and the other in his descending colon at age of 58. After the second colectomy and partial resection of the ileum, he recovered.

### 3.4. III-3: The Proband's Cousin

At the time of study, the proband's elder cousin was 49 years old. She developed colorectal carcinomas at the age of 29. After experiencing intense abdominal pain, she was diagnosed with CRC. Early diagnosis and treatment enabled her to survive and recover.

### 3.5. III-1: The Proband

The proband is a 43-year-old man. He complained of occasional abdominal pain and blood in the stool for 5 years; this was accompanied by a defecation frequency of 5-6 times/day. These symptoms could be relieved after taking oral antiphlogistic medicine. However, aggravation of blood in stool compelled him to consult the physician. The colonoscopy identified 0.5cm×1.0cm polyps with smooth mucosa in the transverse colon, as well as enormous ulcerative neoplasm with necrosis on the surface uplift at 35-40 cm away from the anal margin.

A colonoscopy biopsy confirmed that samples from the transverse colon had chronic inflammation on the mucosa with mild atypical hyperplasia in partial glands and that samples from the descending colon had adenocarcinoma. Physical examination and computed tomography (CT) detected no abnormalities in any other parts of his body.

**Figure 2:**
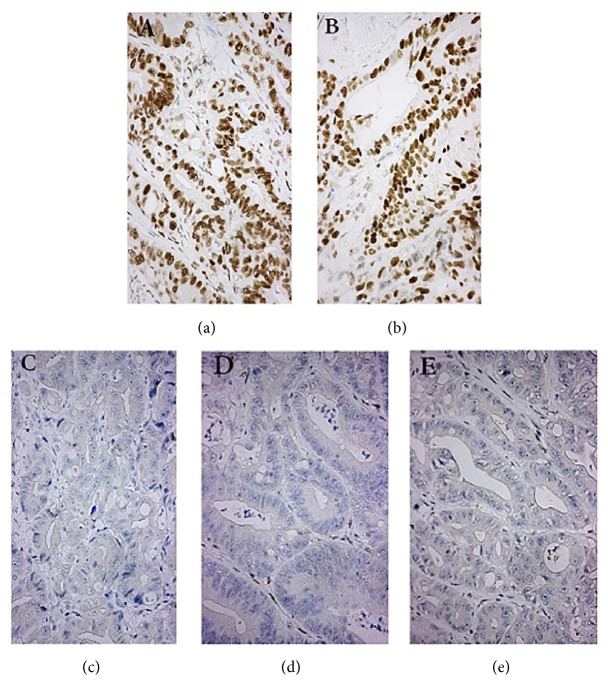
The immunohistochemistry of the proband. (a) represents MSH2, (b) represents MSH6, (c) represents BRAF, (d) represents PMS2, and (e) represents MLH1.

After the left colectomy and adhesiolysis of abdominal cavity, the postoperative pathology revealed a diagnosis of stage pT4aN1M0. The immunohistochemical analysis performed on the proband's tumor tissue identified MSH2 (+), MSH6 (+), BRAF (-), PMS2 (-), and MLH1 (-) (Figure 2). The result of the targeted genetic screening of p.G12D and p.G13D of* KRAS* was negative. According to these tests,* MLH1* has a high probability to be a causative germline mutation in this family.

### 3.6. Sequencing Result

**Figure 3:**
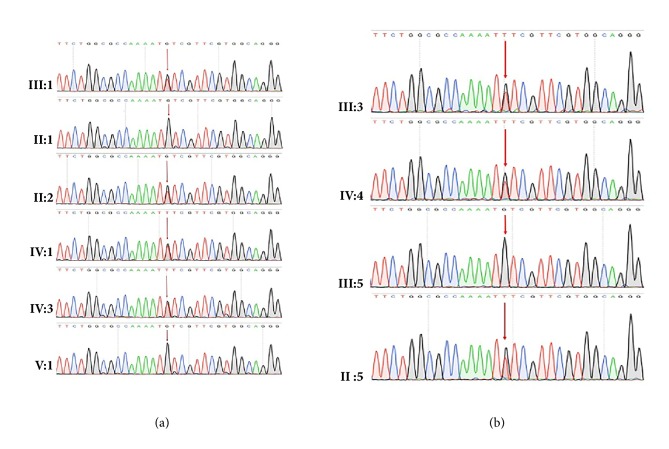
(a) and (b) Sanger sequencing of* MLH1 *(c.3G>T, p.Met1Ile) in these family members identified in the proband and his father (GenBank Accession: NM_000249.3).

Only one novel initiation codon mutation (c.3G>T of* MLH1 *gene) was identified by the genetic screening from both the proband and his father. This mutation was verified by Sanger sequencing using primers: F-5′-AGACCCAGCAACCCACAG-3′, R-5′-TTCCTCCACTTACACTCCAAA-3′. According to the validation, this mutation was confirmed to be cosegregated in this family (Figures 1, 3(a), and 3(b)). This mutation leads to a nonfunctional MLH1 protein, which potentially reduces activity [17].

### 3.7. Real-Time Quantitative RT-PCR Result

**Figure 4:**
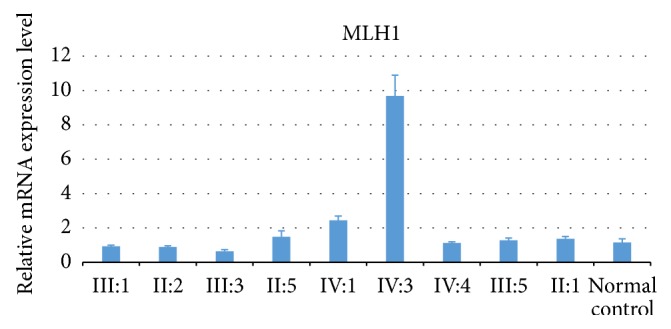
The relative mRNA level of MLH1 in these family members with or without MLH1 mutation. III:1, II:2, and III:3 are patients, II:5, IV:1, and IV:4 are unaffected carriers, III:5 and II:1 are non-carriers in this family, and normal control is a non-carrier of MMRs gene with no blood relationship to this family. The levels of MLH1 in patients were lower than those with wild type MLH1 or unaffected carriers.

The MLH1 mRNA level was measured from 1 normal control and 9 family members which includes the 3 patient carriers, 3 normal carriers, and 3 non-carriers. Target gene qPCR data were normalized using GAPDH as a reference gene. With sample II:2 to be control group, the relative MLH1 mRNA expression value was calculated via the 2^-ΔΔCt^ method and listed in Table 1. A significant difference of MLH1 mRNA level was exhibited between patients and normal family members (including unaffected carriers and normal non-carriers).* MLH1* mRNA levels in patients were lower than normal* MLH1* mutation carriers and normal non-carriers (Figure 4).

### 3.8. Statistics

**Figure 5:**
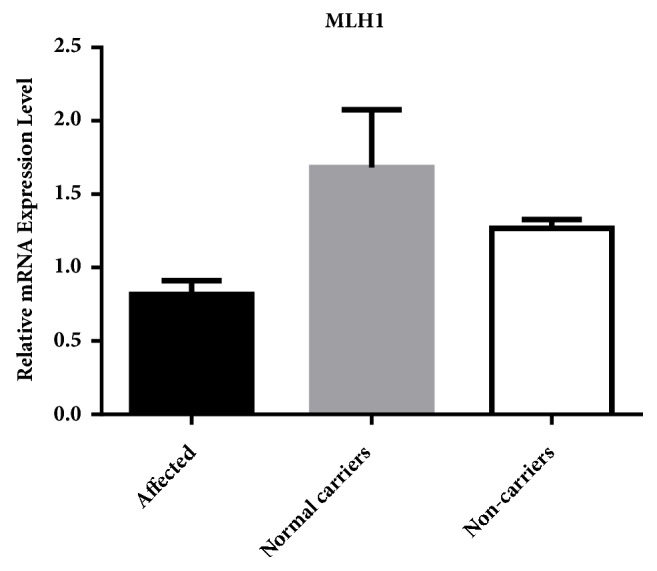
MLH1 RNA average expression level among affected group is much lower than that among normal carriers group (P < 0.05); meanwhile, MLH1 RNA average expression level among affected group is also lower than that among non-carriers group (P < 0.05).

We calculated the P value between the 3 patient carriers and the normal carriers (expect for IV:3 who is still so young, 9 years old, that his mRNA level might influence the average level) and between the 3 patient carriers and the 3 non-carriers, respectively (Figure 5). There is significant difference between the patient carriers and the normal carriers, as well as between the patient carriers and the non-carriers, respectively.

## 4. Discussion

The MLH1 initiation codon mutation (c.3G>T, p.Met1Ile) is a unique and potentially pathogenic MMR mutation detected in this family with Lynch syndrome and is cosegregated among the affected members in this family. It is classified with strong evidence of pathogenicity by American College of Medical Genetics and Genomics (ACMG) standards and guidelines [17]. Some other initiation codon mutations of the* MLH1* gene have been reported in Lynch syndrome families.* In vitro* functional studies of* MLH1* (c.1A>G, p.Met1Val) mutation revealed that the translation is mostly initiated 103 nucleotides downstream, but also at other two ATG sequences downstream. Because this mutation results in a frameshift mutation of start codon, it will lead to formation of truncated proteins. The major transcriptional product showed decreased mismatch repair activity* in vitro* compared to known pathogenic mutations. The other two transcriptomes showed either minimal protein expression (initiated at c.89) or moderate expression (initiated at c.122) (Parsons et al., 2015). Other initiation codon mutation of* MLH1*, (c.2T>A, p.Met1Lys) [18], (c.2T>C, p.Met1Thr) [19], and (c.2T>G, p.Met1Arg) [20, 21], were reported to be pathogenic because of MSI-H and abnormal IHC detected in tumors. The* MLH1* (c.3G>A, p.Met1Ile) mutation was detected in a family with Lynch syndrome [22]. This research provides more evidence of pathogenicity for this* MLH1* (c.3G>T, p.Met1Ile) mutation. Considering this family's pedigree, we speculate that this start codon mutation of* MLH1* is potentially pathogenic in nature; the partial loss of MLH1 mRNA expression indicates that this mutation might be associated with an intermediate penetrance of the Lynch syndrome phenotype.

Recent research of MLH1 mRNA levels in peripheral blood lymphocytes has concentrated on differentiating aspects of hereditary nonpolyposis colorectal cancer from normal controls [23], or determining mutation pathogenicity [24]. Controls in those studies are non-carriers of MMRs mutation. In our present research, we discovered that the carriers of the same* MLH1* mutation showed different mRNA levels in peripheral blood compared to the control group and carriers of the mutation that have not been affected by Lynch syndrome. The mRNA level of MLH1 in carriers that have recovered from colorectal cancer decades ago is still lower than that of unaffected carriers and non-affected mutation carriers. As unaffected carriers have never suffered from HNPCC-related symptoms, the* MLH1* mRNA level is almost 2 times higher than the mutation carriers affected with Lynch syndrome. It should be noted that the relative gene expression of the 9-year-old unaffected carrier IV:3, which is not mentioned in Figure 3, is 12 times more than that of affected carriers. The relative gene expression of unaffected carrier IV:4, who is 27 years old, is lower than the average of the unaffected carriers and non-carriers; close follow-up will be taken on this carrier to make an early diagnosis. No significant relationships were found between mRNA levels in peripheral blood lymphocytes and the early diagnosis of the MMRs mutation carriers; besides the selective transcription of the initiation mutation, there are several other mechanisms which may also influence the expression of MLH1 in different carriers. Epigenetic regulation may play crucial roles to drive carcinogenesis. Promoter methylation of MMRs could lead to reduced protein expression [25]. Histone modifications could regulate the hMLH1 alternative splicing [26]. Some microRNA such as miR-422a could suppress MLH1 expression through base paring with the MLH1 3′-untranslated region. A feedback loop could be formed between the microRNA transcription and the MLH1 expression and controls cell growth and proliferation [27].

## 5. Conclusions

The detection of germline MMRs mutation, protein expression, and MSI [15, 28, 29] is mature and has been applied extensively in clinics. The MMRs mutation could help genetic counselors to predict the lifetime cancer risk of the carriers. The protein expression and MSI of tumor tissues could help doctors to evaluate the survival time as well as enable them to select suitable therapy. The initial purpose of genetic testing and immunohistochemistry was to determine an operation plan and to select appropriate chemotherapy. The carrier situation in this family provides more evidence to the intermediate penetrance of the detected MLH1 (c.3G>T, p.Met1Ile) mutation. The mRNA expression level of MLH1 in the peripheral blood of the family members suggests that it may serve as a biomarker for early diagnosis of Lynch syndrome. In order to determine whether the mRNA level of MMR is normal, the definition of normal range of the mRNA level of MMR depends on long-term follow-up of the unaffected carriers of this family and detection of large scales of similar Lynch syndrome families. Normal controls are also certainly required. Whenever the mRNA level of some unaffected carrier is obviously decreasing, the relative surveillance and detection should be applied. Our research provides a new avenue for a simple noninvasive technique in the early diagnosis of Lynch syndrome. We are planning to expand this study to a larger population to validate whether the mRNA level of MMR in peripheral blood detected by qPCR could be applied to large Chinese cohort.

## Figures and Tables

**Table 1:** The MLH1 mRNA expression level of in the family members.

Family member	Mutation state	CRC state	MLH1 mRNA expression(2-*⊿⊿*Ct)	Average expression (2-*⊿⊿*Ct)
III:1	YES	38	0.80041/1.000/1.000	0.93347
II:2	YES	27	0.77876/0.995/0.887	0.88692
III:3	YES	29	0.74267/0.436/0.719	0.632556667
II:5	YES	no	2.1416/0.973/1.339	1.484533333
IV:1	YES	no	2.7604/1.957/2.606	2.441133333
IV:3	YES	no	9.9197/7.469/11.654	9.6809
IV:4	YES	no	1.2054/0.993/1.163	1.120466667
III:5	NO	no	1.5237/1.166/1.159	1.2829
II:1	NO	no	1.1539/1.635/1.304	1.3643
normal control	NO	no	1/0.895/1.576	1.157

## Data Availability

The datasets generated and/or analyzed during the current study are not publicly available due to the Chinese ethic rules, but are available from the corresponding author on reasonable request.
